# Word learning dogs (*Canis familiaris*) provide an animal model for studying exceptional performance

**DOI:** 10.1038/s41598-021-93581-2

**Published:** 2021-07-07

**Authors:** Claudia Fugazza, Shany Dror, Andrea Sommese, Andrea Temesi, Ádám Miklósi

**Affiliations:** 1 Department of Ethology, Eötvös Loránd University, Budapest, Hungary; 2 MTA-ELTE Comparative Ethology Research Group, Budapest, Hungary

**Keywords:** Psychology, Human behaviour

## Abstract

Exceptional performance is present in various human activities but its origins are debated and challenging to study. We report evidence of exceptional performance and qualitative variation in learning object-names in dogs. 34 naïve family dogs and 6 knowledgeable individuals that knew multiple toy names, found in 2 years of search around the Globe, were exposed to 3 months of training to learn two novel toy-names and were tested in two-way choice tests. Only 1 naïve and all 6 knowledgeable dogs passed the tests. Additionally, only these dogs learned at least 10 new toy names over the 3 months, showing qualitative variation in this capacity. Although previous object-name knowledge could provide an explanation for the superior performance of the knowledgeable dogs, their rarity and the absence of previous training of this skill point to exceptional giftedness in these individuals, providing the basis to establish dogs as a model-species for studying talent.

## Introduction

The origin of behavioural individuality is a fundamental question in cognitive science, neuroscience, psychology, and evolution. Individual variability in external and internal organ morphology is present in all organisms, including among genetically identical individuals, such as human identical twins (e.g.^1^). Individual variability is also present in behaviour and in various domains of cognitive performance^2^. Exceptional performance—i.e., the performance of outliers in individual variability—is a well-known, established phenomenon that is also present in various human activities such as music, mathematic and linguistics (e.g.^3^). Since Galton’s monograph *Hereditary Genius*^4^, the origins of individual variation in cognitive skills have been debated. While some authors suggest that genetic factors play the most significant role^5,6^, others propose a preponderant role of environment and deliberate practice (e.g.^7–11^).

At the population level, cognitive performance in the different domains mostly shows a continuous distribution (e.g.^12^) but there is no consensus on the mechanisms behind individual variation (e.g.^6,10–12^). For example, variation in human mathematical ability is believed to originate from the integration of several different factors such as an extensive network of cognitive skills and mathematics-specific knowledge, underpinned by specific aspects of motivation (e.g.^13,14^). Some rare individuals, however, show an exceptional talent in a specific domain. Their performance represents an outlier in the distribution, and the factors behind this exceptional performance are not known. Albert Einstein and Wolfgang Amadeus Mozart are well known examples of this phenomenon (e.g.^15,16^).

The biological underpinnings of human exceptional performance are extremely challenging to study. For example, mathematical giftedness and its cognitive and neurological correlates were investigated in several studies, but these mostly suffer of low statistical power and methodological issues (^17^ for review), partly because it is difficult to find a relatively large population of exceptionally gifted subjects. Moreover, experimental manipulations which may shed light on the mechanisms and origins of exceptional performance pose obvious ethical concerns making this type of research infeasible on human subjects.

Some cognitive traits seem to show also a qualitative variation. For instance, while in general, musical performance varies continuously and shows a quantitative variation across individuals, absolute pitch, the ability of some musicians to precisely identify and name musical tones without hearing a reference tone^18^, offers an example of a trait that exhibit qualitative rather than quantitative variation. Absolute pitch is considered an ideal model for investigating the influences of genes and environment on the development of a highly specific ability and its neural underpinnings, thus it has stimulated research in neuroscience, psychology genetics and music science^19^. Absolute pitch has been proposed to rely on two main cognitive processes: long-term memory representations of pitches and their association with meaningful labels^20^. Interestingly, recent findings suggest that absolute pitch relies on the size of the auditory cortical area in the brain^21^, suggesting that qualitative differences in a cognitive trait may be supported by quantitative differences at the neural level.

The discovery of individual traits that are stable over time in nonhuman vertebrates (e.g.^22^) and invertebrates can facilitate research on behavioural variation (e.g.^23^). These animal models offer genetic (e.g.^24^), neuromodulatory (e.g.^25^) and neurodevelopmental^26^ explanations for systematic behavioural differences.

Recently, dogs have been proposed as an ideal natural model for the study of several cognitive and behavioural traits that are shared with humans. Dogs are easily available and tractable subjects and, because they evolved and develop in the human environment^27^, they represent a more valid model species, compared to laboratory animals, especially for the socio-cognitive domain. Particularly, in the study of the comparative aspects of the evolution of skills related to faculties of language^28^, the dog offers interesting insights into how some functionally similar traits may emerge in phylogenetically distant species, involving specific brain areas, and also help shedding light on the evolution of these traits in *Homo* (e.g.^29,30^).

The extant literature on word learning in dogs reported only a handful of cases of individual dogs (most of which were Border collies) being able to learn the name of multiple objects (e.g.^31–33^). These studies only included a single subject each and one study included 3 subjects^34^. On the basis of those extremely low N studies, some researchers may assume implicitly that word learning is a cognitive capacity *typically* present in dogs as a species. However, given the rarity of such dogs, despite of intensive efforts to train dogs for object-names (e.g.^35^) one could argue that this skill may surface only in a few exceptional individuals.

The long-term aim of our research is to go beyond single case studies and establish a population of family dogs with skills of learning object-names to study the origins of exceptional performance in a cognitive trait. Thus, we decided to set up a study in which both puppies and adult naïve dogs are systematically and intensively trained for learning at least two object names over a 3-month period, and we used a strictly controlled testing method to assess the dogs’ learning outcome every month from the start of the training (monthly tests). In addition to these naïve family dogs, we also included in the same training and testing program six so called word knowledgeable adult family dogs (WK-dogs, see also “Methods”) that knew the name of at least 15 toys. Before starting the training program, these six dogs participated in a stringent baseline test to establish their knowledge of the names of their toys.

We hypothesized that the ability to learn object-names varies continuously among dogs and that developmental neurological mechanisms, including neurological plasticity during early development (e.g.^36^), may support individual differences in this ability. We predicted that puppies would have some advantage over adult dogs in learning object-names after being exposed to such experience during early development, which contributes to shaping their brain and mental capacities in an optimal way to learn words. We also supposed that, if the training method is successful, the performance of family dogs and knowledgeable dogs with regard to learning new object-names should not differ.

Our results, however, pointed to an unexpected *qualitative*, rather than quantitative variation in the capacity to learn object-names, suggesting that this capacity hardly emerges in typical dogs, irrespectively of the age of the subjects and despite intensive training, while a few rare individuals can rapidly master multiple object names. We suggest that the capacity to learn object-names in dogs shows analogies with exceptional performance (talent) in humans.

## Results

### Baseline test for the WK dogs

All WK dogs were successful above chance in the baseline test (Table 1).

**Table 1:** Results of the Baseline test with all the toys of each WK dog. Each dog is tested on his ability to recognize all toys reportedly known by name. One trial per toy is carried out.

ID	N toys tested	N toys retrieved	p-value	Chance level (%)
Gaia	28	16	< 0.001	6
Max	15	11	< 0.001	8
Nalani	37	34	< 0.001	6
Rico	16	16	< 0.001	8
Squall	20	15	< 0.001	6
Whisky	59	54	< 0.001	6

### Monthly test with two toys

**Figure 1:**
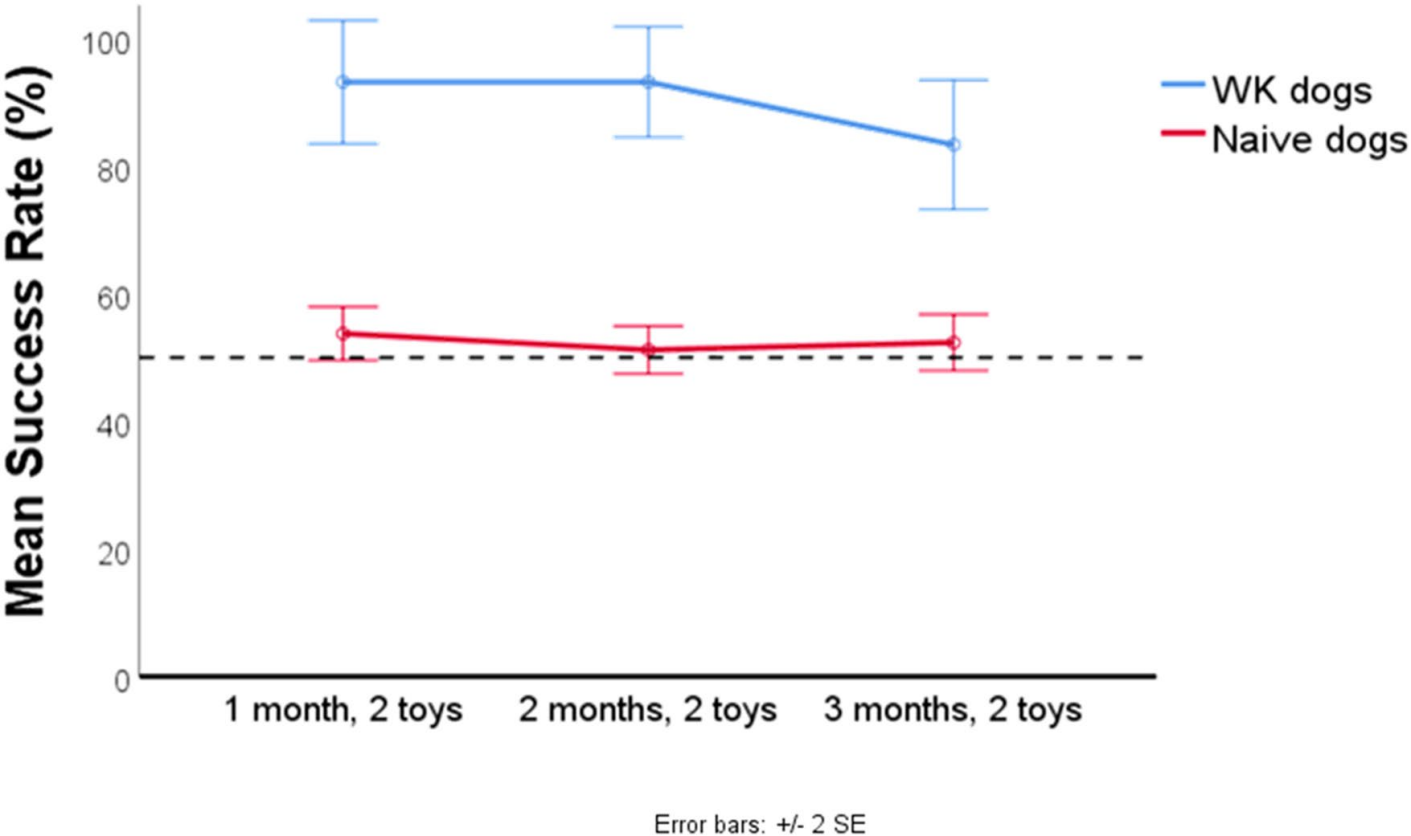
The bar plot shows the overall mean (± SE) success across the 3-months tests of the *naïve dogs*, puppies and adults (first month N = 34, second month N = 33, third month N = 32) and *WK dogs* (N = 6) tested on the two trained toy names in two-way choice tests. Dotted line represents chance level (0.5).

The success of naïve puppies and naïve adults did not present any difference (z = − 0.331, p = 0.941). Hence, we clustered all the naïve subjects together for further analysis. The performance of the two groups (naïve and WK dogs) did not depend on the trial (z = − 1.516, p = 0.129) while there was a significant difference between the performance of the naïve and WK group (z = 8.661, p < 0.001), with the WK dogs being significantly more successful (Fig. 1). The performance of one of the naïve dogs was surprisingly good (100% correct trials, binomial test, p < 0.001, chance level = 0.5). Hence, we performed Grubbs’ test. As expected, the result revealed that this subject, an adult Border collie named Oliva, was an outlier (absolute z-score value = 3.08, threshold at = 2.908). Oliva only participated in the 1st month test (the mean of the group was 54% ± 12.32).

### Monthly test with all new toys learned

Only the WK dogs and Oliva, the subject detected as an outlier in the naïve dogs’ group, participated in these tests because none of the other dogs could successfully learn the name of the first two toys.

**Figure 2:**
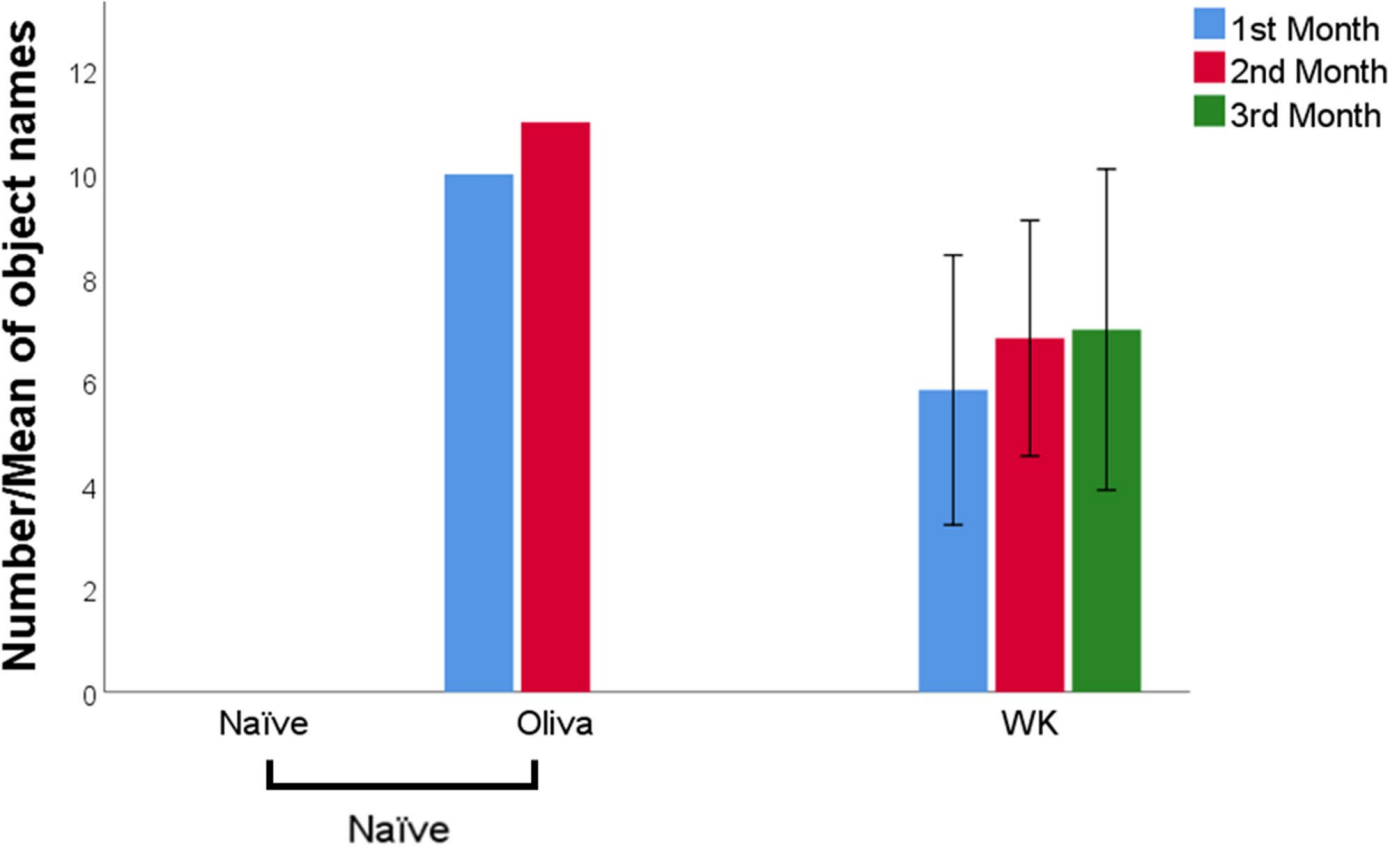
The bars show the number (for Oliva) or the mean ± SE (for all other subjects) of toys learned each month by the two dogs’ groups (naïve and WK) and by the outlier Oliva, the only naïve dog that learned new toy names over the course of the experiment.

The six WK dogs learned between 2–11 (7 ± 3.5) new toy names in the first month; between 3–12 (6.9 ± 3.2) new toy names in the second month, and between 2–13 (7 ± 3.8) new toy names in the third month (Fig. 2).

Oliva learned 10 new objects in the first month (binomial test, p < 0.001, chance level = 0.10) and 11 more in the second month (binomial test, p < 0.001, chance level = 0.09—Fig. 2). She was not tested further because she passed away after this test.

## Discussion

Our results show that, while the vast majority of the naïve dogs, irrespectively of their age, showed very little evidence of learning object-names despite 3 months of unusually intensive exposure and practice, seven individuals (six WK dogs and one naïve dog out of 34) learned multiple object-names very quickly.

These intriguing results strongly point to a *qualitative*, rather than a quantitative individual variation in the capacity of learning object-names in dogs, suggesting that this capacity may represent a special skill, that tends to emerge only in some individuals. These few *gifted word-learner* individuals are outliers and can be considered exceptional, at least with regard to learning names of objects.

This above hypothesis can be challenged on at least the following grounds:

First, the two group of dogs may differ in some non-specific behaviour traits needed for high performance.

We can reasonably exclude that naïve dogs failed to learn because of motivational factors related to the task of retrieving toys. First of all, we had selected puppies and adult dogs that were motivated for fetching toys. Moreover, these subjects did not show any sign of reduced motivation for the task throughout the experiment: they appeared always eager to fetch a toy and they invariably did so in all test and training trials. Yet, they failed in relying on the toy name to select it.

Second, WK dogs had previous experience with learning and knowing object-names. Although, this fact cannot be denied, importantly, Oliva, in the naïve dog group, showed the capacity to learn multiple object-names without having previous experience of object-name learning. Thus, for the emergence of the capacity to learn multiple object-names, previous experience does not seem to be fundamental. Based on our limited number of subjects we cannot not exclude that previous experience may further facilitate rapid learning (see also^39^); however, our results point to this experience not being an indispensable requisite.

Importantly, the owners of the six dogs with previous vocabulary knowledge almost invariably reported that they did not specifically train the dogs to learn toy names. The dogs appeared to have learnt object-names spontaneously during the typical play interactions that occur between most owners and their dogs. Only after the owners realized that the dogs had learned several object-names, some of them started to teach the names of toys on purpose, still through similar play sessions, with the only difference that now the owners were aware that the dog may learn the object names. Thus, it seems that these dogs show an exceptional capacity to learn in the absence of formal training.

The variation of the number of new toy names that the WK dogs learned in a month is most likely due to the different amount of time and number of new toys provided by the owners. However, the most interesting finding is that these dogs *did* learn *several* new toy names, while all but one of the naïve dogs did not show much evidence of learning.

Third, different type of training protocols may have resulted in higher performance in naïve dogs.

We cannot exclude the possibility that different types of training protocols or even more intensive and extensive training could be more effective. Particularly, we cannot exclude that even earlier exposure in puppyhood may lead to different results. However, our training protocol cannot be considered ineffective because at least one naïve subject and the six WK dogs learned multiple object-names by applying it.

In addition, owners may have behaved with their dogs in slightly different ways during exposure while applying the protocol at home and this may have affected the dogs’ ability to learn, but it is unlikely that such small differences would have resulted in the failure of learning even only two toy-names of all naïve dogs but one in the naïve group and success in learning multiple toy-names of all dogs in the other group. Furthermore, training protocols applied by the owners at home are typically successful^35,37,38^. Consistently with our results, Ramos and Mills^35^ showed that typical dogs could successfully learn commands for actions but not for objects.

Fourth, owners of the WK dogs may have been more experienced in dog training. There were no major differences among the owners’ expertise in the two groups. Only one of the owners of WK dogs is a dog trainer and at least 10 owners of naïve dogs were dog trainers or dog experts. Most owners in both groups were not dog trainers.

In addition, during the 3-month long intensive training program all owners received continuous help and encouragement from our team. Despite this, only 30 owners out of 47 completed the training and testing procedure. It is likely that many of those who did not complete it, decided to drop out because they lost motivation by seeing their dogs repeatedly failing in the training and testing.

Moreover, among the dogs that were able to learn new object-names, some (Squall, Oliva, Nalani and Max) lived in a multi-dog household or the owners had had other dogs before and yet, only one of the dogs in the family had shown the ability to learn object-names. We think it is unlikely that dogs living in the same household were exposed to very different treatments and that the owners would behave very differently with them.

All the 7 dogs that were able to learn object names in this study were Border collies. However, it is worth noting that, also in the naïve group, 18 dogs were Border collies and, yet they did not show evidence of learning object names. The prevalence of Border collies over other breeds in this and previous studies provides some suggestive evidence for genetic influences potentially acting at least at two levels: (1) Border collies (or more generally herding breeds), being selected for visual and acoustic interaction with humans, may have some predisposition for this trait; (2) some specific combination of genetic variation may further facilitate the emergence of the capacity for object name learning. However, we also note that the vast majority of Border collies in our study did not show this trait and at least two dogs reported by previous studies to have learned multiple object names are Yorkshire terriers^33,39^, which is not a breed selected for herding.

In summary, it seems that purely environmental, social and other non-specific individual factors (e.g., motivation for toys) do not explain the exceptional performance of the 6 WK and 1 naïve dog. Our results rather point to *talent* as a label for the extremely specific skill of those few individuals. Thus, we suggest that these subjects are *gifted word-learners*, in the sense that they possess a specific quality which is far above the typical population and that their exceptional capacity and performance parallel the phenomenon of talent and extreme individual variation in cognitive traits in humans.

To discuss the extremity of the performance of the dogs that learned multiple object-names, it may be revealing to draw a parallel between the observed phenomenon and absolute pitch in humans.

1. Analogies between the representational skills: Both, gifted humans with absolute pitch and gifted word-learner dogs are able to identify an item based on a label: the name of the note^21^ or the name of the object. Thus, it seems that they are able to mentally classify sounds/object-names into remembered categories. Gifted word-learner dogs, like humans with absolute pitch, may possess a referential mental representation or a template that they use to categorize the objects. Categorization skills in absence of specific training were found in a previous study with one of these dogs, Whisky^30^. Whisky was able to sort novel dog toys into four known categories, without having previously been trained to categorize, but simply having been exposed to a typical dog’s life in a human family.
2. Rarity: People with absolute pitch are rare, with estimates of 1 < in 10,000 persons reported^18^. There are no estimates of the proportion of dogs with the ability to learn object names but in our study, out of 34 dogs motivated for toys, only one (2.94%) showed the capacity to learn toy names. Moreover, this subject was not randomly selected among owners motivated to participate in the study, like the other naïve dogs. Her owner thought that, among her dogs, this one was “special” in the way she would listen and react to human language. Therefore, the above-mentioned 3 out of 100 may well be an overestimation. The rarity of the capacity to learn object-names in dogs is also indirectly corroborated by the fact that the existing studies on object-name learning in dogs always include only one or very few subjects. This is consistent with this capacity being an exceptional trait possessed only by some gifted individuals.
3. Limited effect of training: The capacity to learn object-names did not emerge in most subjects, even after 3 months of intensive training. Similarly, absolute pitch does not emerge in musicians even after tens of thousands of hours practicing and reading scores^40^.
4. Sensitive period: The manifestation of some skills, like first language learning, may rely on mandatory experience during a restricted window of early mental and neural development (e.g.^41^). In the case of absolute pitch there are both supporting^42–44^ and non-supporting^21^ findings whether early experience to musical training is needed for the emergence of this skill. Here we found that intensive exposure during puppyhood is not enough for the emergence of object-name skills in typical dogs. Whether and the extent to which earlier exposure (during a possible sensitive phase) is needed for this talent to emerge remains to be determined.

The exposure to formal training sessions (e.g.^45,46^) is often preferred because the closeness of events and their repetitive occurrences increase the chances for the formation of mental associations, especially in the case of arbitrary tasks. In contrast, during spontaneous interactions it is probably more difficult to recognise similar relationships. Thus, learning in the latter case may rely on partly different mental mechanisms. In addition, gifted word-learner dogs need only a few exposures for learning object-names, as revealed by two dogs with previous vocabulary of multiple object-names^47^. Those two dogs were able to learn new object-names in only four exposures in a social, playful context, providing evidence for rapid mapping (e.g.^48,49^). Taken together, the mental process allowing gifted word-learner dogs to acquire new object-names during spontaneous social interactions, may share some important features with the mental apparatus supporting word learning in young infants^50–52^ during the so-called vocabulary spurt^53^.

We argue that the dog provides an ideal, representative and tractable model for studying the origins and mental mechanisms of qualitative variation in cognitive performance. This study, although exploratory, aimed at taking the fundamental first step in this direction by providing evidence of the existence of this phenomenon in dogs learning object names. Its significance is comparable to exceptional performance in humans, such as absolute pitch and other similar phenomena in music, mathematic, and linguistics.

## Methods

### Subjects

#### Naïve family dogs (naïve dogs)

To test developmental differences in the capacity to learn object-names, we recruited 47 dogs motivated for toys and we enrolled them in an intensive 3 months long training program, aimed at teaching them toy names. The dogs’ motivation for toys was assessed in a preliminary behavioural test during which they were asked to retrieve toys that were laid on the floor three consecutive times. Only the dogs that picked the toys in their mouth all 3 times were recruited.

The owners of the dogs volunteered for the study. 13 owners abandoned the program before reaching the first test. Thus overall, we collected data of 34 dogs, of which 19 adults (mean age: 52.3 months ± 30.8 SD) and 15 puppies (mean age: 3.1 months ± 0.83 SD). 18 of these dogs were Border collies.

All the naïve dogs, apart from four adult Border collies, were recruited in Budapest (Hungary), through the Family Dog Project database. The other four dogs were recruited in Brazil. Two of them were relatives of Gaia, one of the WK dogs (see below) and one of them belonged to the same owner as one of the relatives. These dogs were recruited through Gaia’s owner. The fourth naïve dog from Brazil, Oliva, was recruited as the owner came to know about this project and asked to participate because she believed that one of her two Border collies had an uncommon predisposition to listen to human speech, although she did not have previous knowledge of object-names. Oliva’s health was compromised since puppyhood and we assume that she was not exposed to play sessions with toys very often for this reason.

#### Word knowledgeable dogs (WK dogs)

Parallelly, we searched around the Globe, through intensive social media announcements, for dogs reported by their owners to know the name of their toys.

We found and recruited N = 6 dogs, all Border collies (mean age in months 43.7, SD 32.3), that could select their toys based on their name. Whisky reportedly knew N = 59 toy names, Max N = 15, Rico N = 16, Nalani N = 37, Gaia N = 28 and Squall N = 20 toy names.

To categorize an individual as a WK dogs, his knowledge of the name of his toys was assessed in a baseline test (see below) and the dog was included in this category only if s/he succeeded in this test above chance.

### Baseline for WK dogs

Before being introduced in the training program described below, the WK dogs were tested in a Baseline test during which we assessed their knowledge of the name of all their toys. During the test, the owner asked the dog to fetch the toys one by one, on a randomized basis, from an out-of-view area, while the dogs’ toys were laid on the floor of the adjacent area—thus out of view from the owner, to control for potential inadvertent visual cues directing the dog to the correct toy. In every trial the dog was requested to choose from 16–20 randomly determined toys for dogs Gaia, Nalani and Whisky; 12–15 toys for Max and 12–16 for Rico (because they only knew respectively 15 and 16 toy names). In every trial, the owner typically asked the dog to bring the toy pronouncing its name “Bring <object-name>!”.

After every 5 trials (or after every 4 trials for Max) another set of 5 randomly chosen toys was taken to the out-of-view area, so that the number of toys the dogs could choose from always varied from 16 to 20 or from 12 to 15/16. The number of trials varied for each dog based on how many toy names it reportedly knew.

The dogs, upon hearing the owner pronouncing the name of a toy, typically went to the out-of-view area, selected a toy and brought it to the owner. The toy that the dogs had in their mouth upon appearing in the area where the owner was, was considered as the selected toy.

If the dogs made a mistake, the owner repeated the request, but this repeated trial was not considered in the analysis of the results.

### Training procedure

During our search for the WK dogs we inquired with the owners about how they had trained their dogs for this skill. The owners reported that they had not specifically trained their dogs. The dogs appeared to have learnt the names spontaneously during unplanned, non-systematic playful interactions. This was also similar to the report of how Whisky (included in this study) and Vicky Nina (not included because she had passed away) had learned the name of multiple toys^43^. Thus, the general description of the playful interaction formed the basis of our training procedure for learning about names of novel toys. This procedure is also similar to what was reported in previous studies on word learning dogs (e.g.^31,33^).

All dogs (naïve and WK dogs) were exposed to an intensive program constituted by multiple approximately 5–10 min long daily play sessions with toys, during which the owners were asked to repeatedly pronounce the name of the toy. The owners were requested to show the toy to their dogs while pronouncing its name and then to play with it by giving it to the dog, playing tug of war, tossing it and asking the dog to fetch it, always repeatedly saying its name. Dogs received a brief play session with the fetched toy for choosing the named one. They were also rewarded by praise, and / or food (depending on each individual dog motivation). Dogs that were rewarded solely with playing with the fetched toy and dogs that were also receiving food as reward were present in both groups. The toys were introduced gradually, one at a time. If the owners and trainer noticed that the dogs were able to discriminate between two toys based on their names, more toys were introduced, always one by one.

During the 3 months, the owners were individually instructed in a training session every week by one of the authors, who is also an experienced dog trainer (SD) and continued the training on a daily basis at home. The weekly training sessions in which the trainer instructed the owners, took place either at the Department of Ethology at ELTE University or during Skype sessions, due to both the spread of the COVID-19 pandemic and the distance of some of the dogs that lived in other countries.

### Performance tests

#### Monthly tests with 2 toys

To be able to compare the dogs’ performance, we tested all dogs (naïve dogs and WK dogs) after 1, 2 and 3 months from the start of the training program, on their ability to discriminate between only the first two toys introduced to them. The testing protocol was similar to the one described for the Baseline test, but only two toys were present. The position of the two toys was semi-randomized so that the same toy was not placed in the same position for more than two times in a row. The two toys were requested by pronouncing their names on a semi randomized basis, so that the same toy was not requested more than two times in a row. After every trial, the toy that the dog selected was carried back, so that there were always two toys to choose from. This test consisted of 12 trials, six for each toy.

#### Monthly test with all new toys

Every month, the dogs that successfully learned the two toy names, were also tested on all the new toys that were introduced by their owners since the start of the program. This test was identical to the Baseline test described above, but only the newly learned toys were present.

Only 7 subjects (1 adult naïve dog, Oliva, and all 6 WK dogs) were included in this test because only those succeeded in learning 2 toy names, so that more toys could be added to their training.

In the 1st month test the new toys introduced by the owners were 11 for Gaia, 7 for Max, 2 for Whisky, 4 for Squall, 6 for Nalani, 4 for Rico and 10 for Oliva.

In the 2nd month test the toys were 23 for Gaia, 14 for Max, 8 for Whisky, 11 for Squall, 12 for Nalani, 8 for Rico and 21 for Oliva (also the new toys learned in the first month are included).

In the 3rd month test the toys were 37 for Gaia, 21 for Max, 16 for Whisky, 19 for Squall, 16 for Nalani and 10 for Rico (also the new toys learned in the first and second month are included). Oliva could not be tested because she passed away.

Of the 47 naïve dogs recruited, 34 continued the training program until being tested at least in the first month test.

34 Naïve dogs and all 6 WK dogs completed the first month test.

33 Naïve dogs and all 6 WK dogs completed the second month test.

32 Naïve dogs and all 6 WK dogs completed the third month test.

### Data analysis

#### Baseline for WK dogs

The statistics were made in R environment (R Core Team, 2016). To analyze the WK dogs’ performance in the Baseline test at the individual level we used the binomial test; chance level was based on how many toys were available for the dogs to choose from. The total number of toys available ranged between 16 and 20 (Gaia, Nalani, Squall, and Whisky) or 12 and 15/16 (Max and Rico). Hence, we set chance level conservatively respectively at 0.06 or 0.08 (Chance level was determined conservatively considering the lowest number of toys on the floor; N = 12 for Max and Rico, and N = 16 for Gaia, Nalani, Squall and Whisky).

#### Difference between age groups in the monthly tests with two toys

For the monthly tests with two toys, the binary outcomes of the two-way choice test were analyzed in binomial generalized linear mixed models (GLMM; glmer function of R package^54^). We compared the success rate of our subjects across age groups; since the performance of the two naïve groups (puppies vs adult dogs) did not differ (see “Results”), we pooled naïve subjects together. We also tested if there was any effect of the trial and found none, therefore we dropped this variable for further analysis.

#### WK and naïve dogs’ performance on the monthly test with two toys

Binomial GLMM was used to compare the performance of our subjects across two groups: naïve and WK dogs. The performance of one of the naïve dogs presented unexpected characteristics, therefore, to detect outliers, we used Grubbs’ test^55^.

#### Monthly test with all new toys learned

To analyze the individual dogs’ performance in the tests with all the newly learned toys we used the binomial test, setting chance level based on how many toys were available for the dogs to choose from.

### Ethical statements

All methods were carried out in accordance with relevant guidelines and regulations for both owners as volunteers and their dogs participating in the experiments.

All experimental protocols for participation of both dogs and their human owners were approved by the National Animal Experimentation Ethics Committee (PE/EA/691-5/2019).

We obtained written informed consent from all dog owners that participated in the study (all above 18 years old).

The study was carried out in compliance with the ARRIVE guidelines.
